# Supplementary material for: A genetically encoded fluorescent biosensor for extracellular l-lactate
Source: Nat Commun. 2021 Dec 6;12:7058. doi: 10.1038/s41467-021-27332-2 (PMC8648760; doi:10.1038/s41467-021-27332-2)
Supplement: Supplementary file 1 — Supplementary Information [file 41467_2021_27332_MOESM1_ESM.pdf]

Supplementary information

A genetically encoded fluorescent biosensor for extracellular L-lactate

Yusuke Nasu, Ciaran Murphy-Royal, Yurong Wen, Jordan Haidey, Rosana S. Molina, Abhi Aggarwal, Shuce Zhang, Yuki Kamijo, Marie-Eve Paquet, Kaspar Podgorski, Mikhail Drobizhev, Jaideep S. Bains, M. Joanne Lemieux, Grant R. Gordon, Robert E. Campbell.

Figure	
Supplementary Figure 1	Construction of the biosensor prototype.
Supplementary Figure 2	Biosensor prototypes based on the various TTHA0766 homologues.
Supplementary Figure 3	Sequence alignment of TTHA0766, cpGFP, and eLACCO1.
Supplementary Figure 4	*In vitro* characterization of eLACCO1.
Supplementary Figure 5	Crystal structure of eLACCO1.
Supplementary Figure 6	Affinity tuning of eLACCO1.
Supplementary Figure 7	*In vitro* characterization of deLACCO.
Supplementary Figure 8	Membrane trafficking of eLACCO1.1 with various leader sequences.
Supplementary Figure 9	Ratiometric imaging of eLACCO1.1 on live HeLa cells.
Supplementary Figure 10	Ca^{2+} titration on live HeLa cells.
Supplementary Figure 11	pH titration on live HeLa cells.
Supplementary Figure 12	Stopped-flow analysis of eLACCO1 and eLACCO1.1.
Supplementary Figure 13	Attempted imaging of extracellular L-lactate with Laconic.
Supplementary Figure 14	Attempted imaging of eLACCO1.1-expressing T98G cells treated with iodoacetate.
Table	
Supplementary Table 1	Crystallographic and refinement statistics of eLACCO1.
Reference	
Supplementary References	
Note	
Supplementary Note	PDB X-ray Structure Validation Report

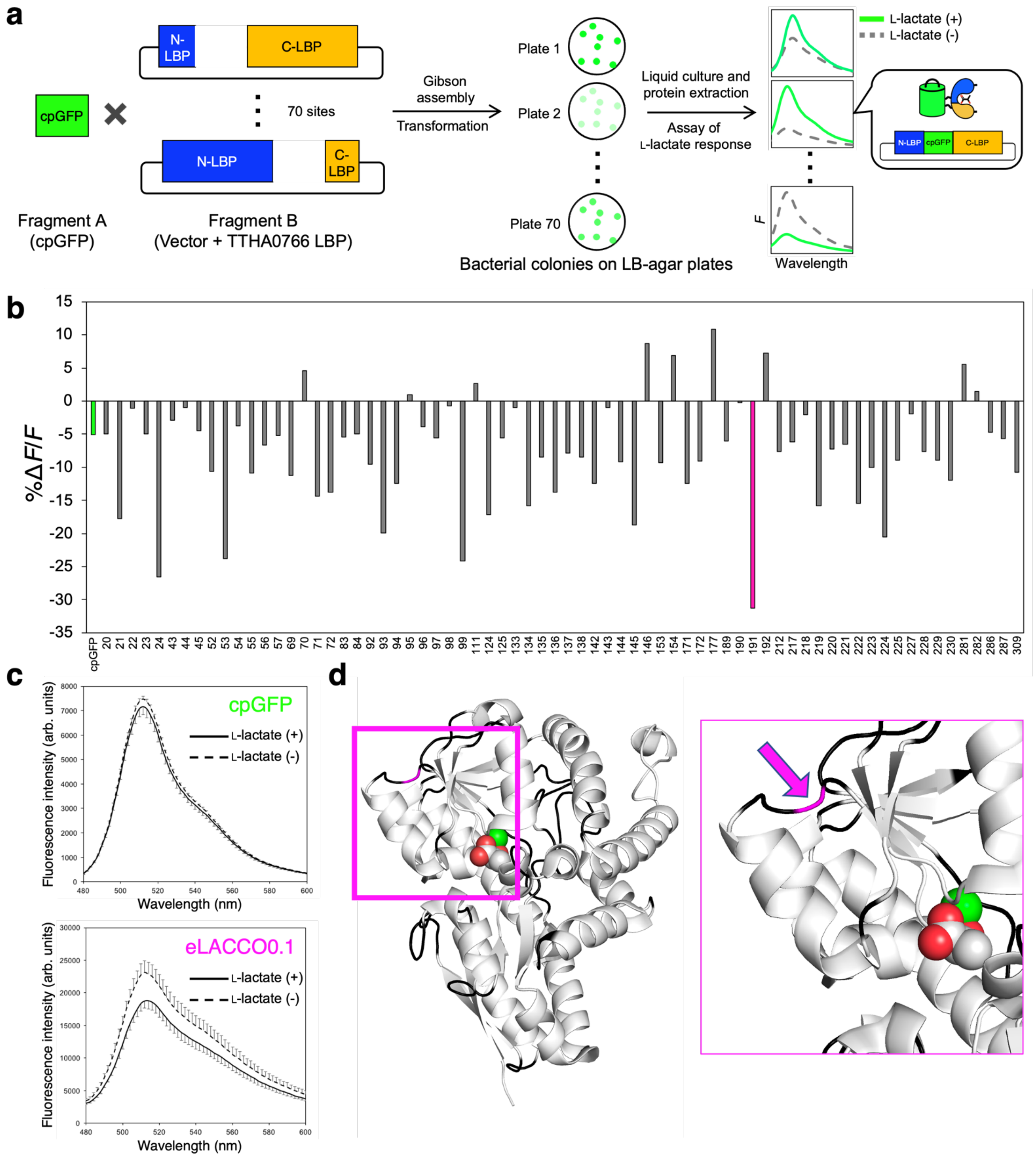

Supplementary Figure 1. Construction of the biosensor prototype.

(**a**) Schematic illustration of the biosensor prototype construction. TTHA0766 L-lactate binding protein (LBP) genes with a range of split sites were ligated with cpGFP gene by Gibson assembly. Transformation of *E. coli* with assembled plasmids resulted in colonies expressing each biosensor prototype protein. Fluorescent colonies were cultured followed by extraction of the protein, and then the change in fluorescence intensity upon 10 mM L-lactate (Δ*F*/*F*) was tested. (**b**) Δ*F*/*F* profile of each candidate protein for prototype biosensor. The number of horizontal axis represents the cpGFP

insertion site on TTHA0766. Δ*F*/*F* of cpGFP as a negative control is shown in green. Red bar at the insertion site 191 showed the largest absolute value of Δ*F*/*F* and the protein was designated eLACCO0.1. (**c**) Emission spectra of cpGFP and eLACCO0.1 in the presence and absence of 10 mM L-lactate. Error bars represent standard deviation of triplicates (mean ± s.d.). (**d**) Crystal structure of TTHA0766 (PDB ID 2ZZV). All insertion sites tested are colored in black. The insertion site 191 is highlighted in magenta.

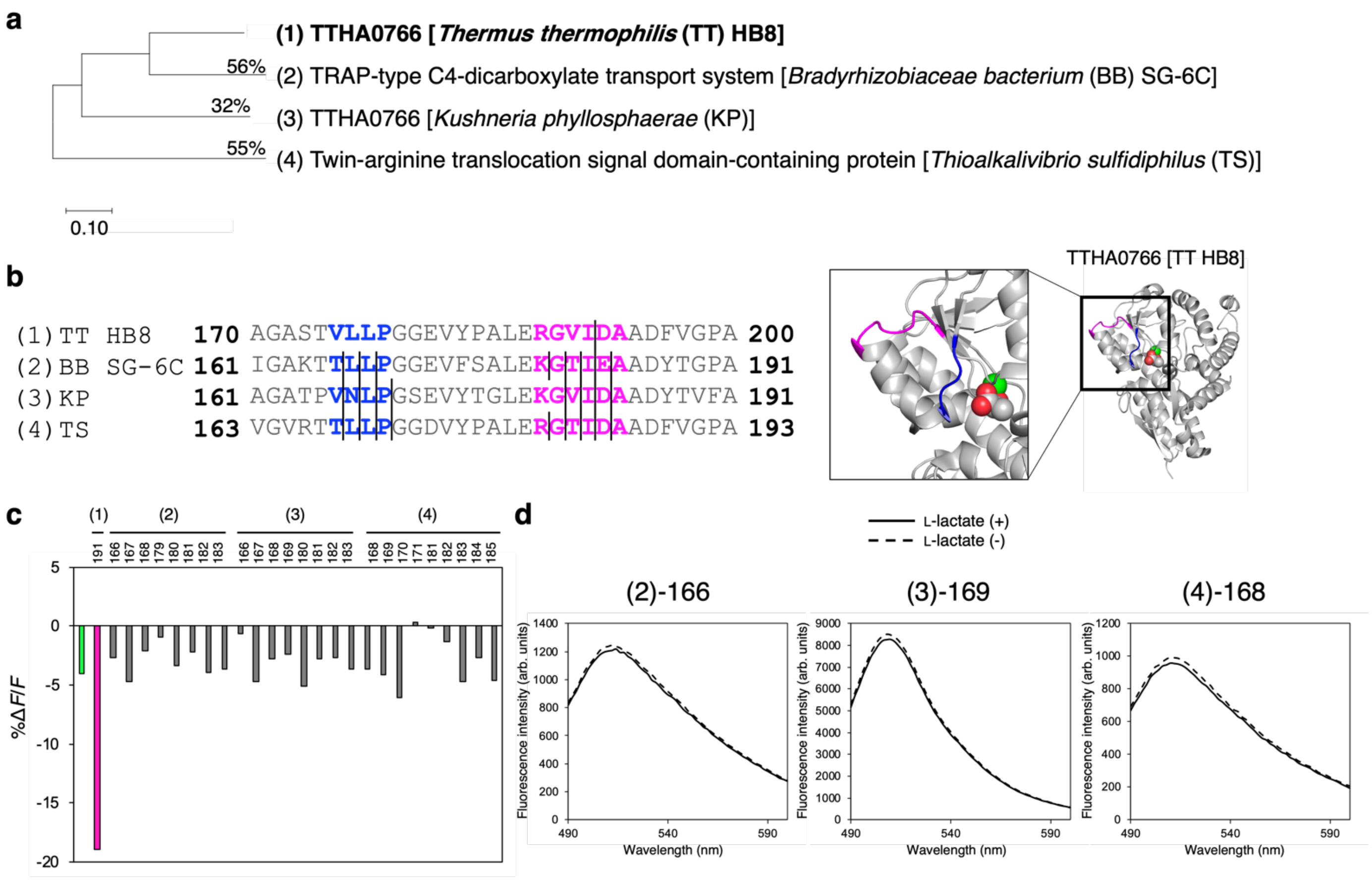

Supplementary Figure 2. Biosensor prototypes based on the various TTHA0766 homologues. (**a**) Phylogenic tree of the various TTHA0766 homologues. Sequence identities of the whole gene in amino acid with *Thermus thermophilis* TTHA0766 are shown as percentages. (**b**) Alignment of the four sequences in the vicinity of the insertion sites. Predicted loop regions from the crystal structure of *Thermus thermophilis* TTHA0766 (right) are highlighted in blue and magenta. Lines represent the sites for cpGFP insertion. (**c**) Δ*F*/*F* profile of each candidate protein for prototype biosensor. The numbers along the horizontal axis represent the cpGFP insertion site on each target protein. Δ*F*/*F* of cpGFP as a negative control and eLACCO0.1 are colored in green and magenta, respectively. (**d**) Representative emission spectra of the prototype biosensor proteins in the presence and absence of 10 mM L-lactate.

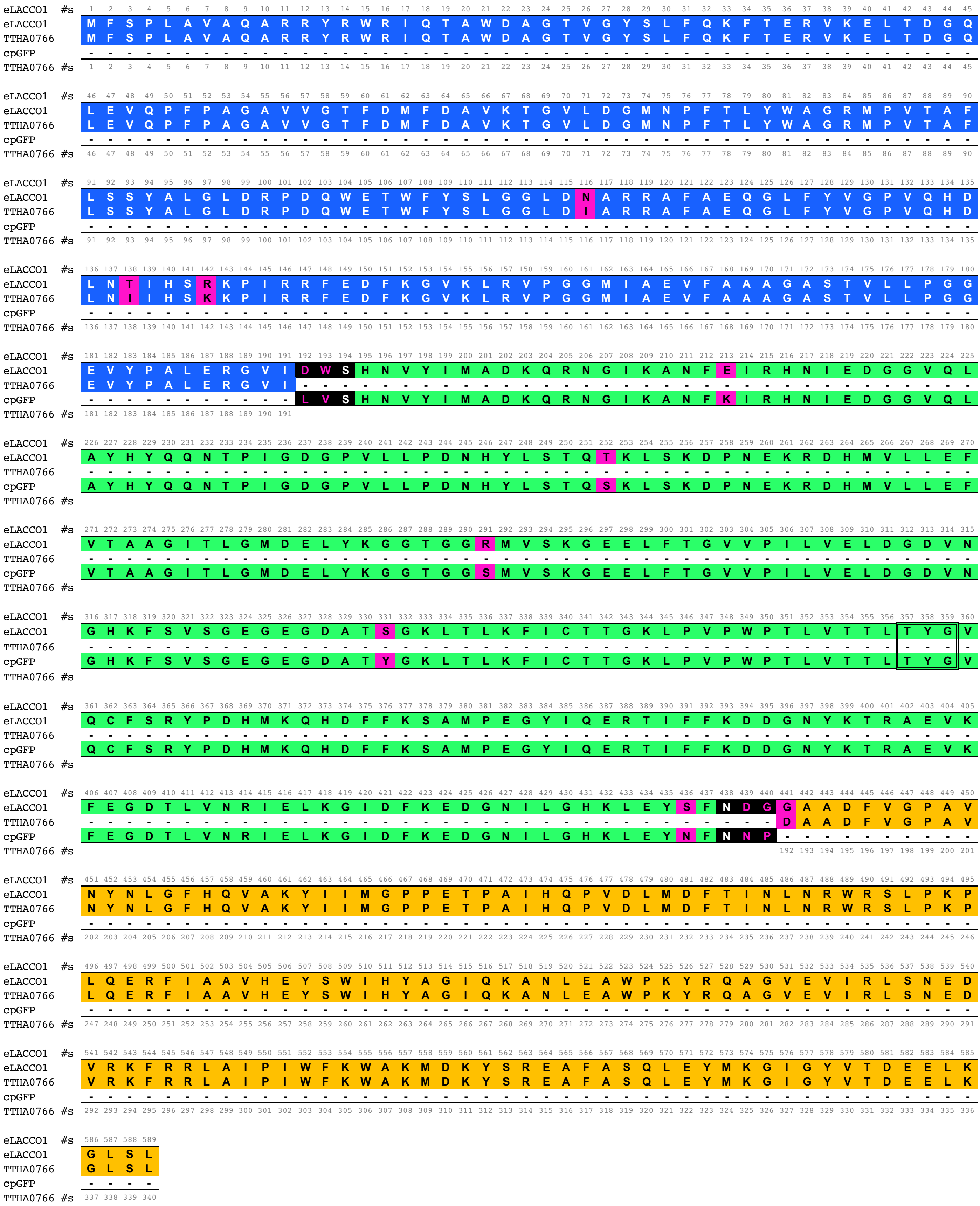

Supplementary Figure 3. Sequence alignment of TTHA0766, cpGFP, and eLACCO1. Mutations in eLACCO1, relative to TTHA0766 and cpGFP, are highlighted in magenta. The chromophore-forming residues are surrounded by a double line.

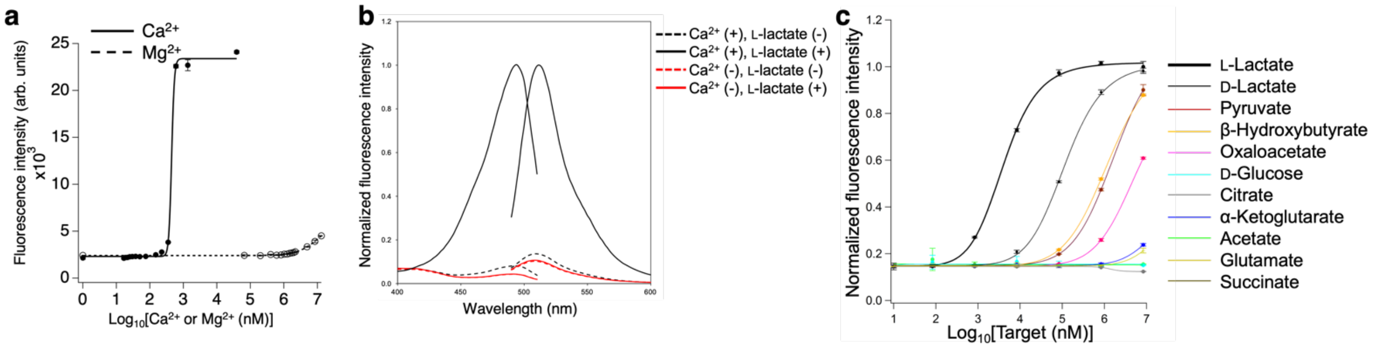

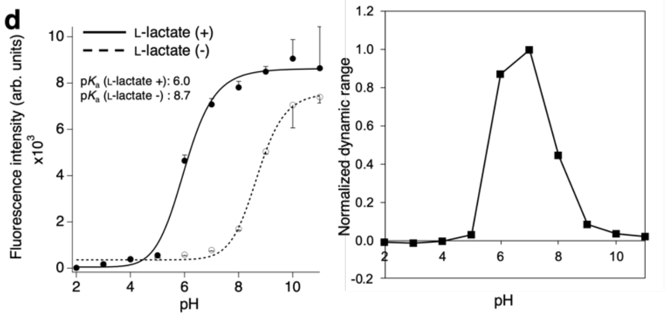

Target	Apparent K_d	Hill coefficient
L-Lactate	4.1 µM	0.94
D-Lactate	120 µM	1.04
Pyruvate	1.9 mM	1.08
β-Hydroxybutyrate	1.3 mM	1.06
Oxaloacetate	5.4 mM	1.14
D-Glucose	n.d.	n.d.
Citrate	n.d.	n.d.
α-Ketoglutarate	n.d.	n.d.
Acetate	n.d.	n.d.
Glutamate	n.d.	n.d.
Succinate	n.d.	n.d.

Supplementary Figure 4. ***In vitro*** **characterization of eLACCO1.**

(**a**) Fluorescence of eLACCO1 in the presence of 10 mM L-lactate as a function of Ca^{2+} and magnesium ion (Mg^{2+}). n = 3 independent experiments (mean ± s.d.). The crystal structure of TTHA0766 reveals an integral and essential Ca^{2+} in the L-lactate binding pocket[1], indicating that L-lactate binding should be Ca^{2+} dependent. (**b**) Excitation and emission spectra of eLACCO1 in the presence and absence of 10 mM L-lactate and 39 µM Ca^{2+}. (**c**) Dose-response curves of eLACCO1 for L-lactate and a variety of metabolites. n = 3 independent experiments (mean ± s.d.). Table summarizes apparent K_d and Hill coefficient for each target molecule. n.d., not determined. (**d**) pH titration curves of eLACCO1 in the presence and absence of 10 mM L-lactate. n = 3 independent experiments (mean ± s.d.).

a

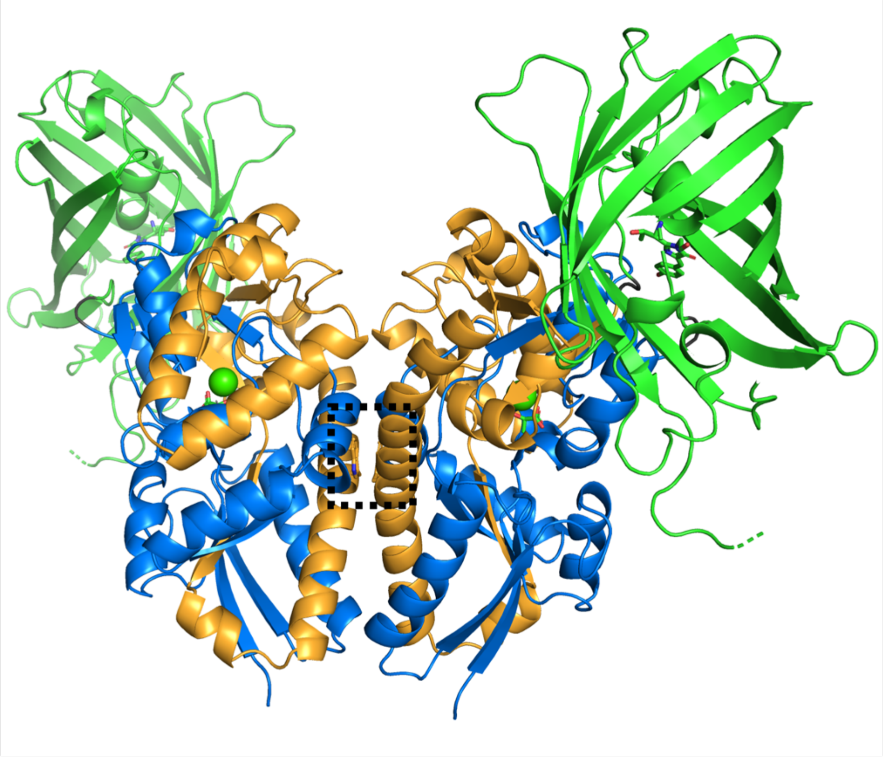

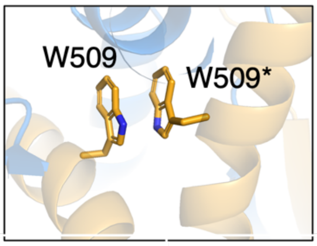

Residue at 509	ΔF/F
W (eLACCO1)	6
K	n.d.
R	n.d.
A	n.d.
L	n.d.
F	n.d.
Y	n.d.
H	n.d.
G	n.d.
T	n.d.
D	n.d.
E	n.d.
S	n.d.

b

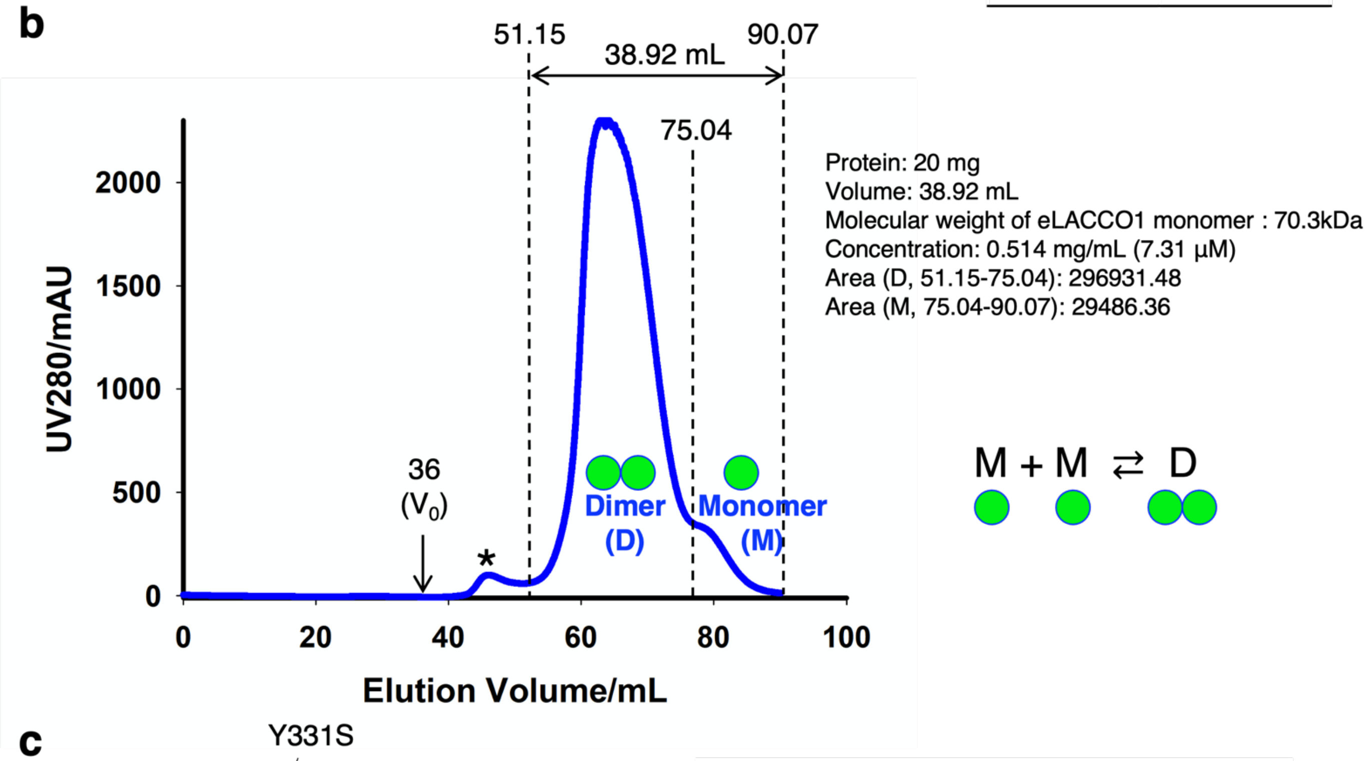

c

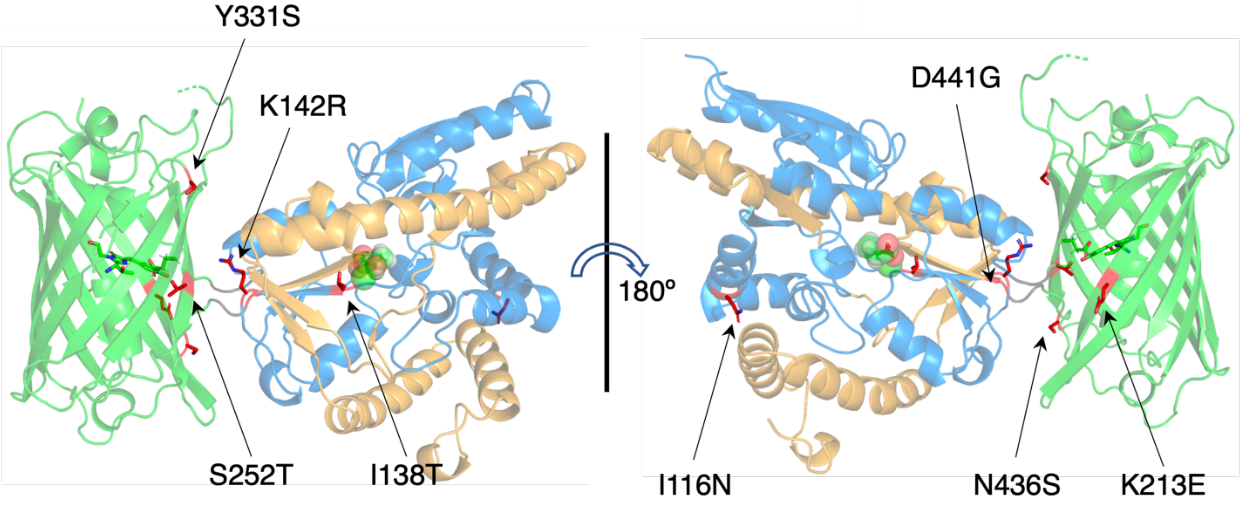

Supplementary Figure 5. Crystal structure of eLACCO1.

(**a**) Dimer structure of eLACCO1. W509 residues of each protomer form a stabilizing electrostatic interaction via π-π stacking. In an effort to disrupt the dimer interface and make the protein monomeric, we performed site-directed mutagenesis on W509. L-Lactate is represented in stick format. Green spheres represent Ca^{2+}. Table summarizes $\Delta F/F$ of W509 variants. n.d., not detected.

(**b**) Size exclusion chromatography of eLACCO1 and estimation of affinity of eLACCO1-eLACCO1 interaction. V_0, void volume of the column. The peak indicated by asterisk is attributed to aggregated proteins derived from sample preparation. The analysis of chromatograph resulted in an estimated dissociation constant (K_d) of 66 nM.

$$[D]/[M] = 296931.48/29486.36 = 10.07$$
$$[D] + [M] = 7.31$$
$$\therefore [M] = 0.660\ \mu M\ (660\ nM)$$

$$\begin{aligned} \boldsymbol{K_d} &= [M]^2/[D] \\ &= [M]^2/10.07[M] \\ &= [M]/10.07 \\ &= 660/10.07 \\ &= \mathbf{66\ nM} \end{aligned}$$

(**c**) Overall representation of the eLACCO1 crystal structure with the position of mutations indicated. L-Lactate is shown in a sphere representation.

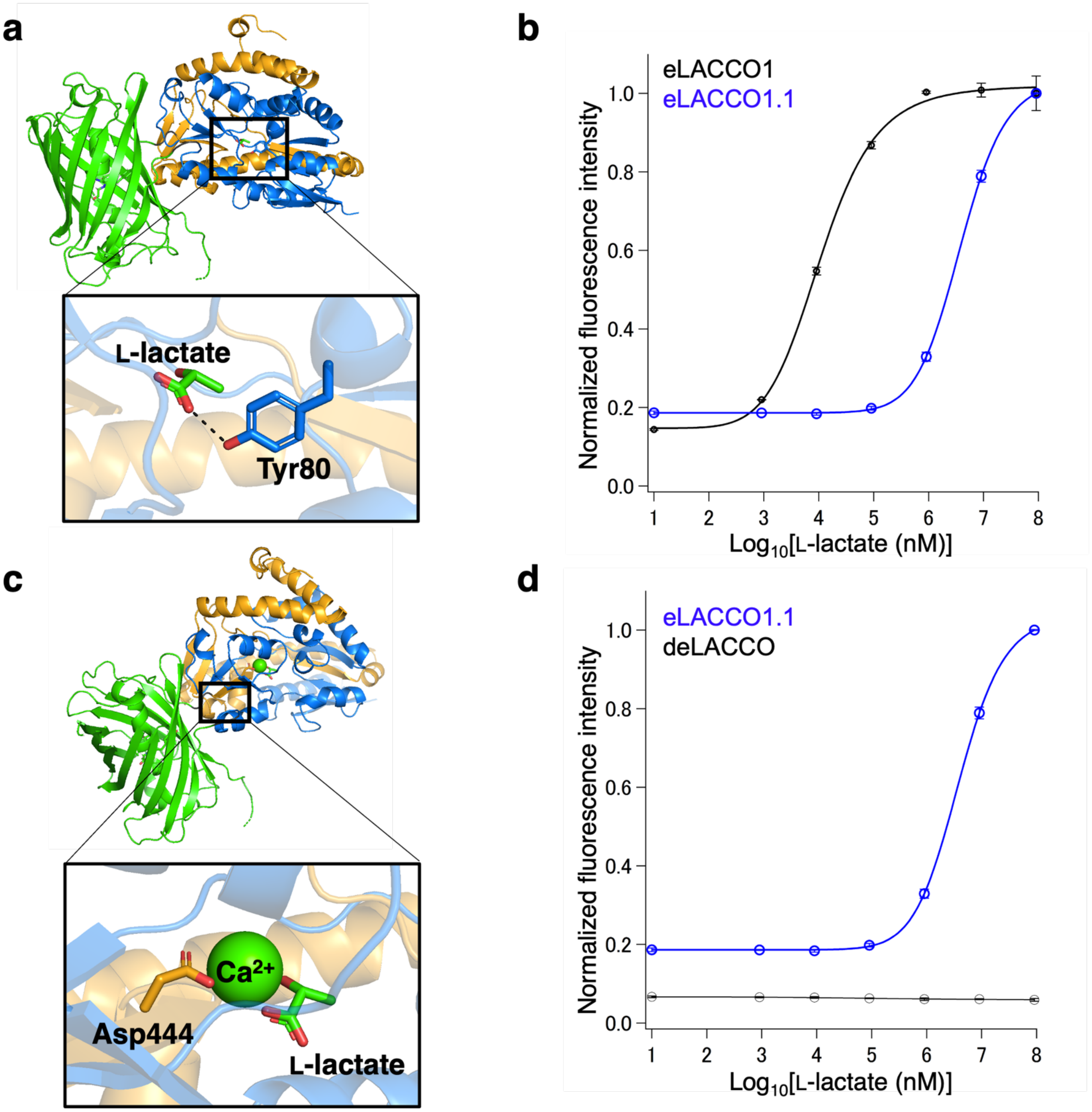

Supplementary Figure 6. Affinity tuning of eLACCO1.

(**a**) Crystal structure of eLACCO1 and zoom-in view of the L-lactate binding pocket. The phenol group of the Tyr80 side chain forms a hydrogen bond to the carboxylate group of L-lactate. (**b**) Fluorescence of eLACCO1 and eLACCO1.1 (eLACCO1 Y80F) as a function of L-lactate. n = 3 independent experiments (mean ± s.d.). (**c**) Crystal structure of eLACCO1 and zoom-in view of the Ca^{2+} binding pocket. Carboxyl group of Asp444 side chain coordinates to Ca^{2+}. (**d**) Fluorescence of eLACCO1.1 and deLACCO (eLACCO1.1 D444N) as a function of L-lactate. n = 3 independent experiments (mean ± s.d.).

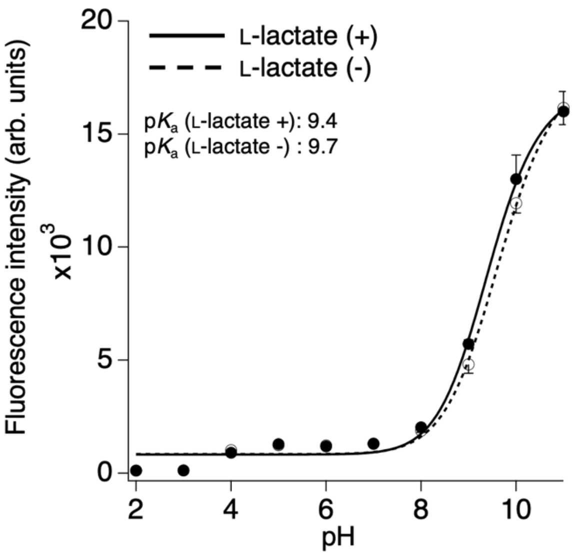

Supplementary Figure 7. ***In vitro*** **characterization of deLACCO.**

pH titration curves of deLACCO in the presence and absence of 100 mM L-lactate. $n = 3$ independent experiments (mean ± s.d.).

a

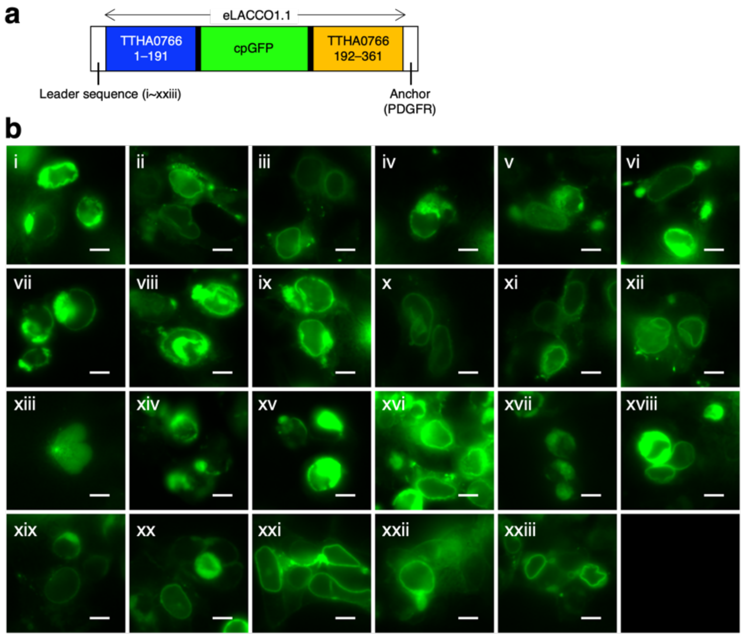

c

	Origin	Species	N-terminal leader sequence
i	Immunoglobulin κ-chain	*Mus musculus*	METDTLLLWVLLLWVPGSTGD
ii	Insulin	*Homo sapiens*	MALWMRLLPLLALLALWGPDPAAA
iii	SPARC	*Homo sapiens*	MRAWIFFLLCLAGRALA
iv	Vesicular Stomatitis Virus G	-	MLSYLIFALAVSPILG
v	Preprochymotrypsinogen	*Homo sapiens*	MAFLWLLSCWALLGTTFG
vi	Interleukin-2	*Homo sapiens*	MYRMQLLSCIALSLALVTNSM
vii	Gaussia luciferase	*Gaussia princeps*	MGVKVLFALICIAVAEA
viii	Albumin	*Homo sapiens*	MKWVTFISLLFLFSSAYS
ix	Influenza Hemagglutinin	-	MKTIIALSYIFCLVLG
x	Frizzled-7	*Mus musculus*	MRGPGTAASHSPLGLCALVLALLGALPTDTRA
xi	CD59	*Homo sapiens*	MGIQGGSVLFGLLLVLAVFCHSGHS
xii	Integrin β pat-3	*Caenorhabditis elegans*	MPPSTSLLLLAALLPFALPASDWKTGEVT
xiii	Calreticulin	*Mus musculus*	MLLSVPLLLGLLGLAAAD
xiv	5HT3A serotonin receptor	*Mus musculus*	MRLCIPQVLLALFLSMLTGPGEG
xv	IgK light chain precursor	*Homo sapiens*	MDMRAPAGIFGFLLVLFPGYRS
xvi	Immunoglobulin heavy chain	*Homo sapiens*	MDWTWRVFCLLAVTPGAHP
xvii	Immunoglobulin light chain	*Homo sapiens*	MAWSPLFLTLITHCAGSWA
xviii	Azurocidin preproprotein	*Homo sapiens*	MTRLTVLALLAGLLASSRA
xix	Cystatin-S precursor	*Homo sapiens*	MARPLCTLLLLMATLAGALA
xx	A conotoxin lp1.3	*Conus leopardus*	MGMRMMFIMFMLVVLATTVVS
xxi	Aspartic proteinase nepenthesin-1	*Nepenthes alata*	MASSLYSFLLALSIVYIFVAPTHS
xxii	Acid chitinase	*Nepenthes rafflesiana*	MKTHYSSAILPILTLFVFLSINPSHG
xxiii	Cholera toxin	*Vibrio cholerae*	MIKLKFGVFFTVLLSSAYA

Supplementary Figure 8. **Membrane trafficking of eLACCO1.1 with various leader sequences.** (**a**) Schematic of the primary sequence of eLACCO1.1 with N-terminal leader sequence and C-terminal PDGFR transmembrane anchor. (**b**) Localization of PDGFR-anchored eLACCO1.1 with different leader sequences in HEK293FT cells. We screened a range of leader sequences in combination with the PDGFR anchor, but did not discover any that led to robust membrane localization of PDGFR-anchored eLACCO1.1. Note that the widely used combination of immunoglobulin κ-chain (Igκ) leader sequence and platelet-derived growth factor receptor (PDGFR) transmembrane domain (corresponding to i) for cell surface expression, resulted in only intracellular expression. Similar results were observed in more than 10 cells. Scale bars, 10 µm. (**c**) Amino acid sequences of the leader peptide tested.

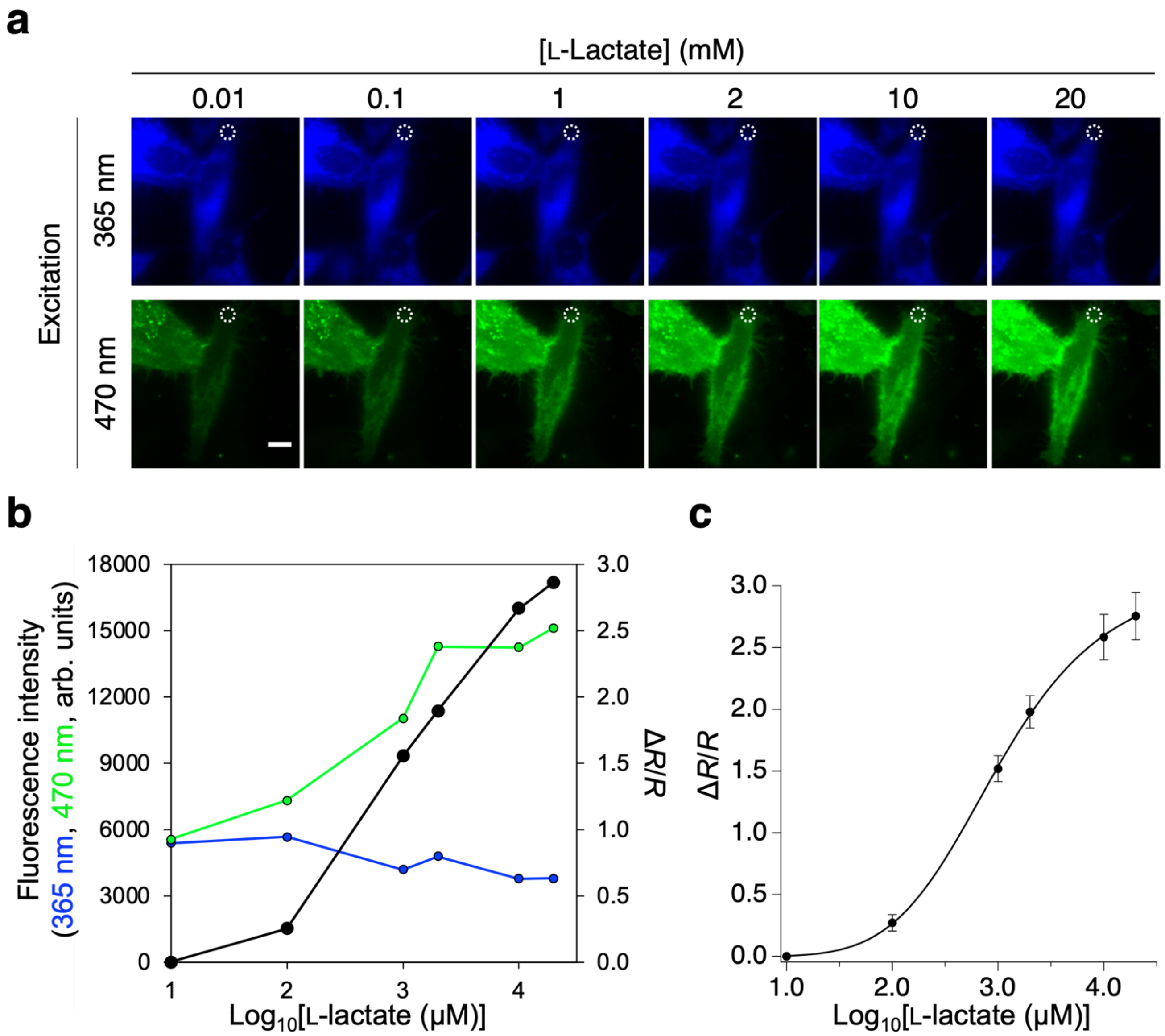

Supplementary Figure 9. **Ratiometric imaging of eLACCO1.1 on live HeLa cells.**

(**a**) Representative images of HeLa cells expressing eLACCO1.1 and treated with different L-lactate concentrations during excitation at either 365 nm or 470 nm. Similar results were observed from three independent experiments. Scale bar, 10 μm. (**b**) Representative trace of fluorescence intensities and Δ*R*/*R* in the region of interest indicated with a dashed white line in (a). (**c**) Δ*R*/*R* of eLACCO1.1 over a range of L-lactate concentrations. Data were fitted with Hill equation. *n* = 8 cells (mean ± s.e.m.).

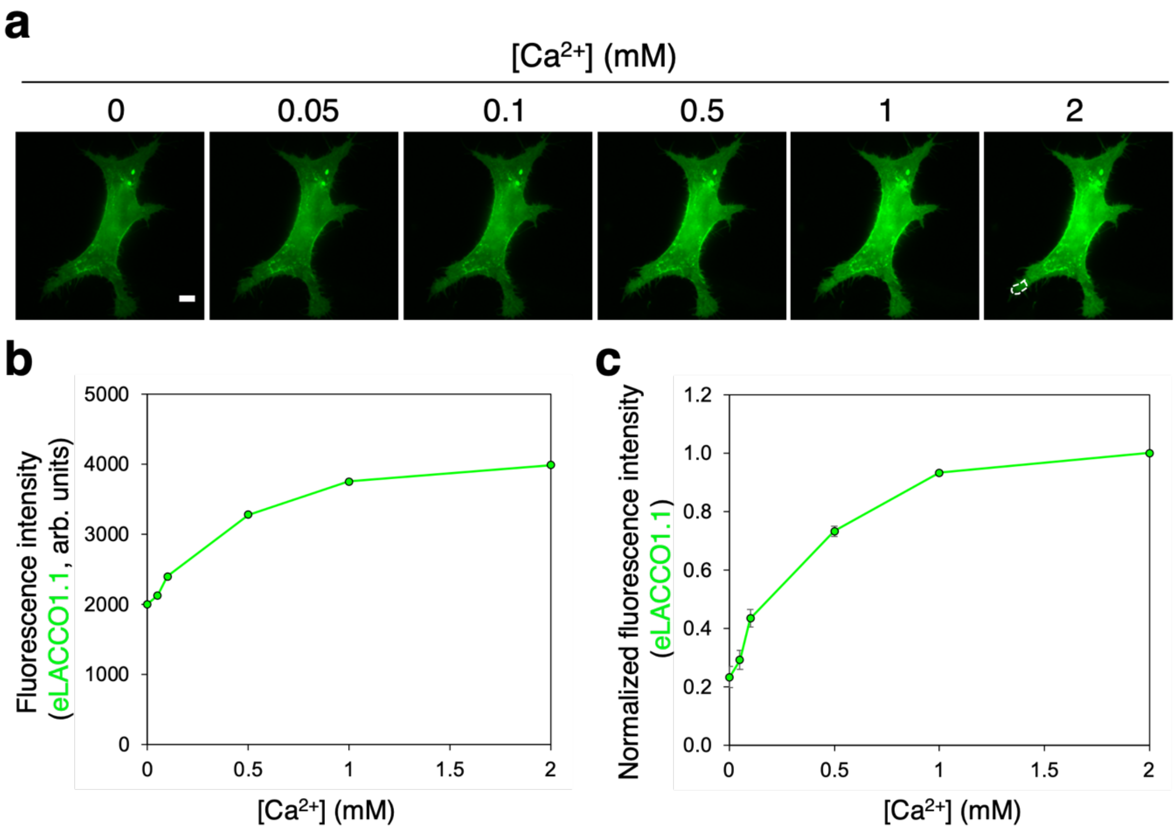

Supplementary Figure 10. Ca^{2+} titration on live HeLa cells.

(**a**) Representative images of HeLa cell expressing eLACCO1.1 at different Ca^{2+} concentrations in the presence of 10 mM L-lactate. Scale bar, 10 µm. (**b**) Representative trace of fluorescence intensity in the region of interest indicated by the dashed white line in (a). (**c**) Normalized fluorescence intensity of eLACCO1.1 at a range of Ca^{2+} concentration. $n = 9$ cells (mean ± s.e.m.).

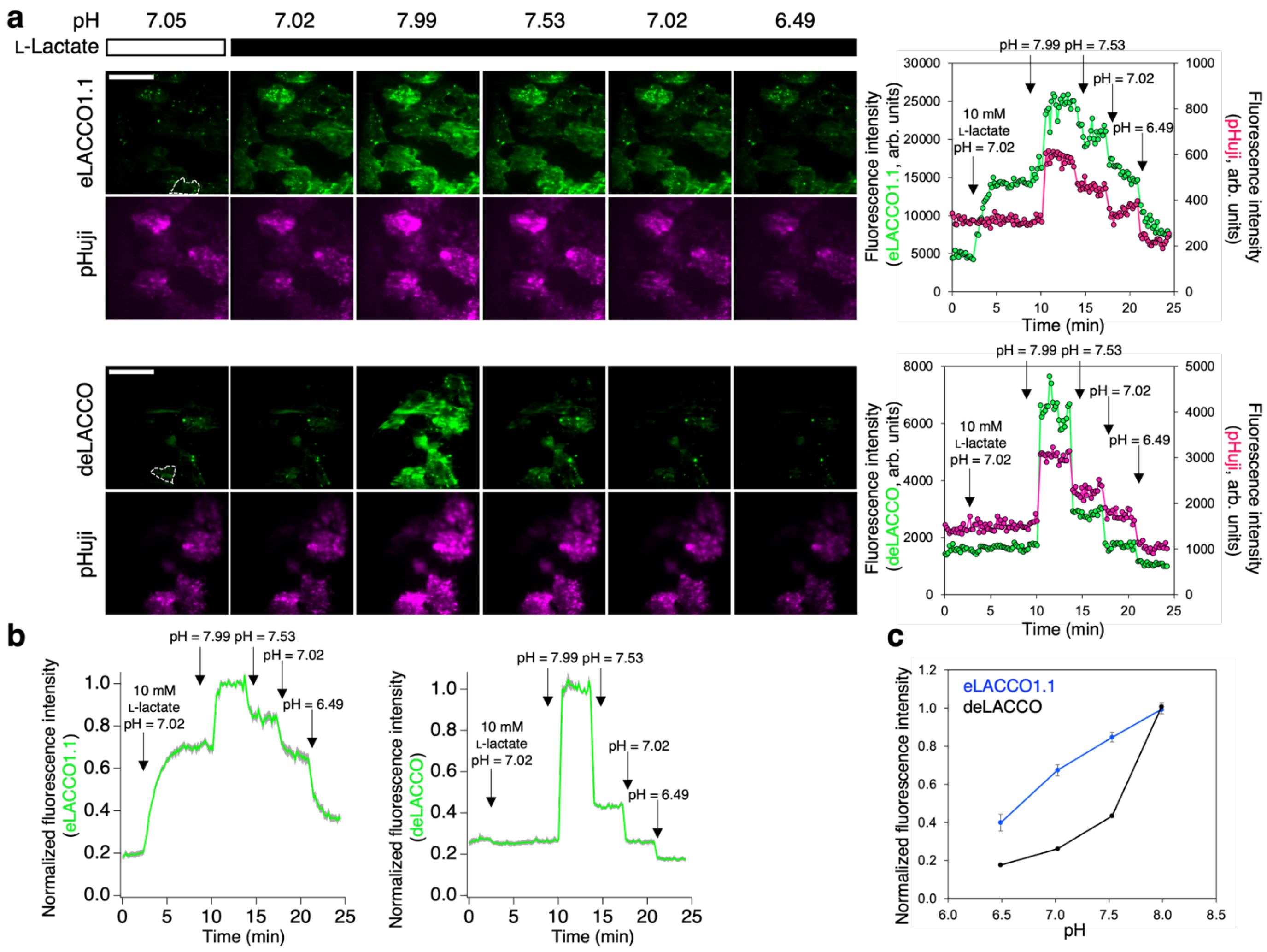

Supplementary Figure 11. pH titration on live HeLa cells.

(**a**) Representative images of HeLa cells expressing eLACCO1.1 or deLACCO and exposed to different pH values. A genetically encoded red fluorescent pH indicator pHuji was simultaneously expressed on the cell surface[2]. Representative traces of fluorescence intensity (right) correspond to dashed white lines on the images. Scale bars, 50 µm. (**b**) The time course of the normalized fluorescence intensity of eLACCO1.1 or deLACCO. n = 29 and 23 cells for eLACCO1.1 and deLACCO, respectively (mean ± s.e.m.). (**c**) pH titration curves of eLACCO1.1 and deLACCO in the presence of 10 mM L-lactate. n = 20 data points at each pH from (b) (mean ± s.d.).

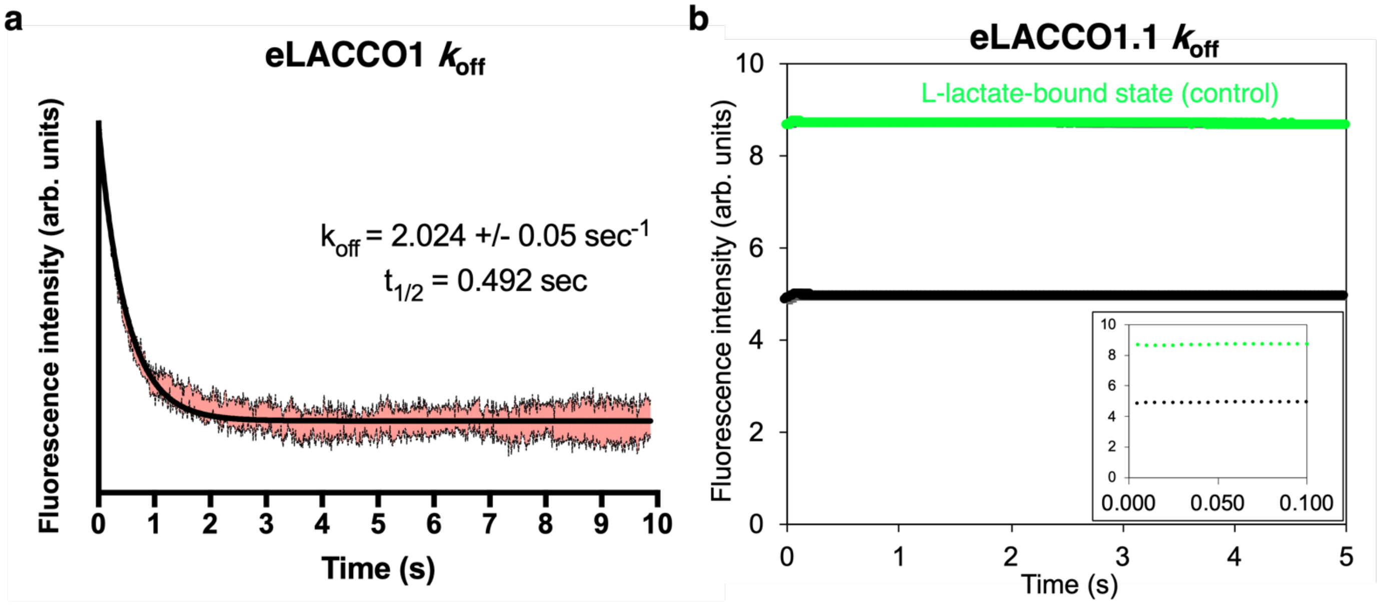

Supplementary Figure 12. Stopped-flow analysis of eLACCO1 and eLACCO1.1.

(**a**) Stopped-flow characterization of purified eLACCO1. L-Lactate-free medium was rapidly mixed at t = 0. $n = 5$ independent experiments (mean ± s.e.m.). (**b**) Stopped-flow characterization of purified eLACCO1.1. L-Lactate-free medium was rapidly mixed at t = 0 (black filled circle). Green filled circles represent the fluorescence intensity of L-lactate-bound eLACCO1.1 as a negative control. Inset is the same data at the time < 0.1 s. $n = 3$ independent experiments (mean ± s.e.m.).

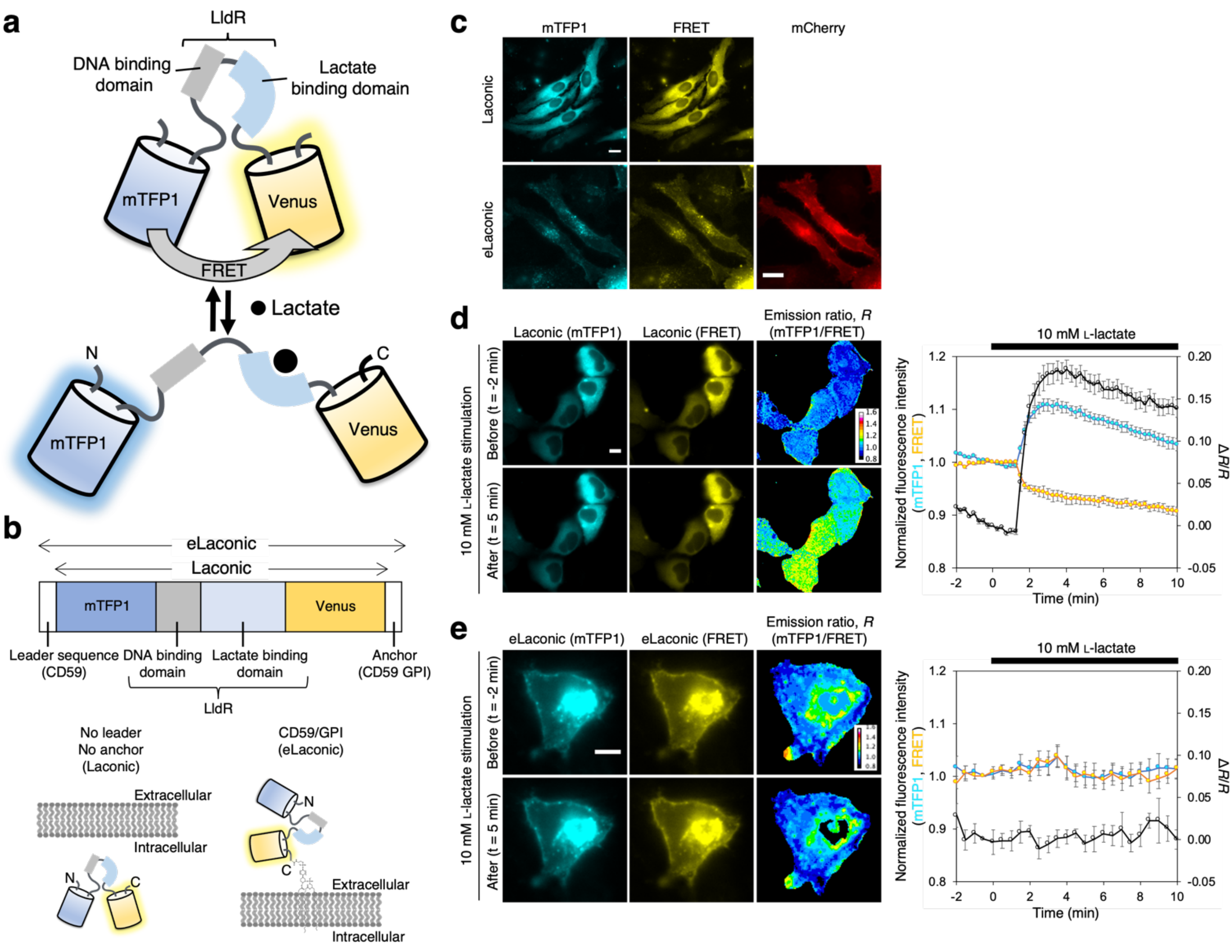

Supplementary Figure 13. Attempted imaging of extracellular L-lactate with Laconic.

(**a**) Schematic representation of Laconic. Binding of L-lactate to LldR L-lactate/DNA binding domain decreases FRET efficiency, thereby changing the fluorescence intensities of mTFP1 cyan fluorescent protein and Venus yellow fluorescent protein[3]. (**b**) Schematic of the primary sequence of Laconic. To directly compare the performance of eLACCO1.1 with the existing L-lactate biosensor, we attempted to target Laconic to the surface of HeLa cells. We named Laconic with N-terminal leader sequence and C-terminal anchor domain as eLaconic. (**c**) Representative images of HeLa cells expressing Laconic or eLaconic. mCherry with Ig*κ* leader sequence and PDGFR transmembrane domain was used as cell surface marker. We found that eLaconic (Laconic fused with the leader and GPI anchor sequences from CD59) could indeed be targeted to the cell surface. Similar results were observed in more than 10 cells. Scale bars, 20 µm. (**d**) Representative images of HeLa cells expressing Laconic before and after 10 mM L-lactate stimulation. HeLa cells were pre-incubated for 1 hour with iodoacetate (500 µM) to block glycolysis at GAPDH, and then imaged in the presence of H^+/K^+ exchanger nigericine (10 µM) and rotenone (2 µM) to equilibrate the extracellular and intracellular L-lactate[3]. Right graph represents the time course of the fluorescence intensity (mTFP1 and FRET) and $\Delta R/R$, where R is the emission ratio (mTFP1/FRET). n = 8 cells

(mean ± s.e.m.). Scale bar, 10 µm. (**e**) Representative images of HeLa cell expressing eLaconic before and after 10 mM L-lactate stimulation. Right graph represents the time course of the fluorescence intensity (mTFP1 and FRET) and $\Delta R/R$. When using treatments similar to those used for imaging of eLACCO1.1, we were unable to observe fluorescent responses from eLaconic. n = 13 cells (mean ± s.e.m.). Scale bar, 10 µm.

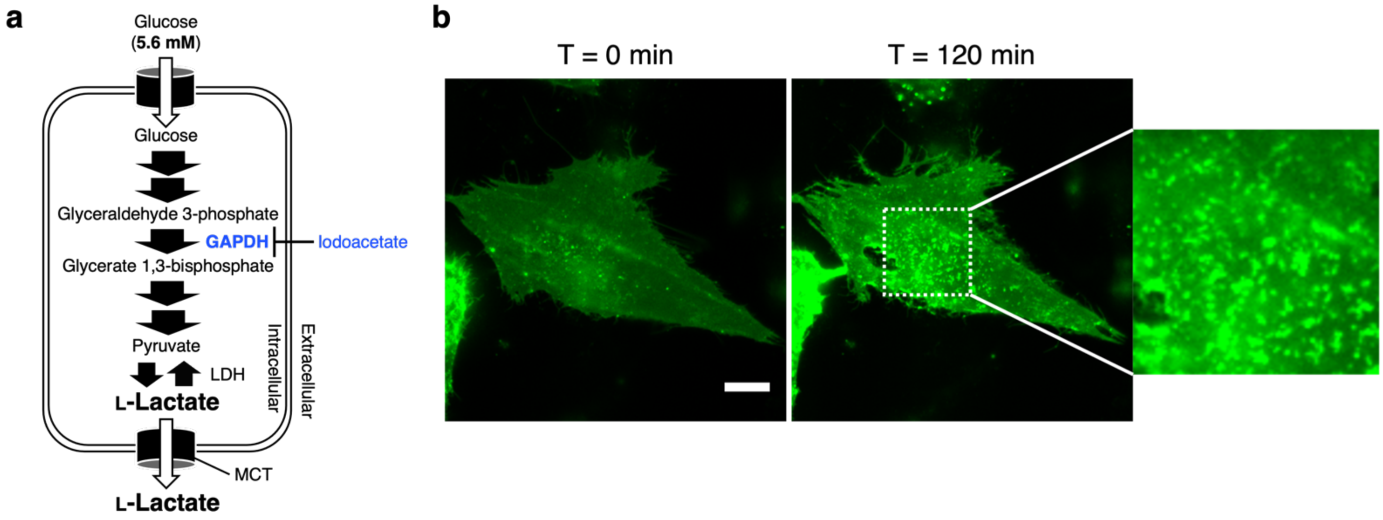

Supplementary Figure 14. Attempted imaging of eLACCO1.1-expressing T98G cells treated with iodoacetate.

(**a**) Schematic representation of L-lactate metabolism in the presence of 5.6 mM of extracellular glucose. GAPDH, glyceraldehyde 3-phosphate dehydrogenase. (**b**) Representative fluorescence images of T98G cells expressing eLACCO1.1 and treated with 100 µM iodoacetate. Iodoacetate modifies cysteine residues via alkylation. eLACCO1.1 has cysteine residues at residue numbers 340 and 362 (Supplementary Fig. 3), and we suspect that iodoacetate might induce aggregation due to the modification of these residues. Scale bar, 20 µm.

Supplementary Table 1. Crystallographic and refinement statistics of eLACCO1.

Crystal	eLACCO1
**Data collection**	
Space group	$C222_1$
a, *b*, *c* (Å)	102.2, 105.0, 138.7
α, β, γ (°)	90.0, 90.0, 90
Resolution (Å)	52.49-2.25 (2.33-2.25)
R_{merge}	0.132 (1.14)
R_{meas}	0.158 (1.3)
Multiplicity	5.3 (5.2)
CC(1/2)	0.986 (0.57)
CC*	0.997 (0.75)
I/σ(I)	5.75 (1.0)
Completeness (%)	99.93 (99.97)
Wilson B-factor (Å^2)	19.33
**Refinement**	
Total Reflections	187301 (18505)
Unique Reflections	35639 (3530)
R_{work}/R_{free}	0.1484/0.1871 (0.1557/0.2109)
**Number of atoms**	
Protein	4497
Ligands	29
Water	511
Average B-factor (Å^2)	21.66
Protein ADP (Å^2)	20.87
Ligands (Å^2)	17.46
Water	28.87
**Ramachandran plot**	
Favored/Allowed (%)	97/3
**Root-Mean-Square-Deviations:**	
Bond lengths (Å)	0.012
Bond Angle (°)	1.44
PDB code	7E9Y

Statistics for the highest resolution shell are shown in parentheses.

Supplementary References

1. Akiyama, N., Takeda, K. & Miki, K., *J. Mol. Biol.* **392,** 559–565 (2009).
2. Shen, Y., Rosendale, M., Campbell, R. E., & Perrais, D. *J. Cell Biol.* **207,** 419–432 (2014).
3. San Martin, A. *et al*. *PLoS ONE* **8**, e57712 (2013).

Supplementary Note

WORLDWIDE PDB PROTEIN DATA BANK

Full wwPDB X-ray Structure Validation Report (i)

Mar 11, 2021 – 04:01 PM JST

PDB ID	:	7E9Y
Title	:	Crystal structure of eLACCO1
Deposited on	:	2021-03-05
Resolution	:	2.25 Å(reported)

This is a Full wwPDB X-ray Structure Validation Report.

This report is produced by the wwPDB biocuration pipeline after annotation of the structure.

We welcome your comments at *validation@mail.wwpdb.org*
A user guide is available at
https://www.wwpdb.org/validation/2017/XrayValidationReportHelp
with specific help available everywhere you see the (i) symbol.

CONFIDENTIAL VALIDATION REPORT

The following versions of software and data (see references (i)) were used in the production of this report:

MolProbity	:	4.02b-467
Mogul	:	1.8.5 (274361), CSD as541be (2020)
Xtriage (Phenix)	:	1.13
EDS	:	2.17.1
Percentile statistics	:	20191225.v01 (using entries in the PDB archive December 25th 2019)
Refmac	:	5.8.0158
CCP4	:	7.0.044 (Gargrove)
Ideal geometry (proteins)	:	Engh & Huber (2001)
Ideal geometry (DNA, RNA)	:	Parkinson et al. (1996)
Validation Pipeline (wwPDB-VP)	:	2.17.1

1 Overall quality at a glance ⓘ

The following experimental techniques were used to determine the structure:
X-RAY DIFFRACTION

The reported resolution of this entry is 2.25 Å.

Percentile scores (ranging between 0-100) for global validation metrics of the entry are shown in the following graphic. The table shows the number of entries on which the scores are based.

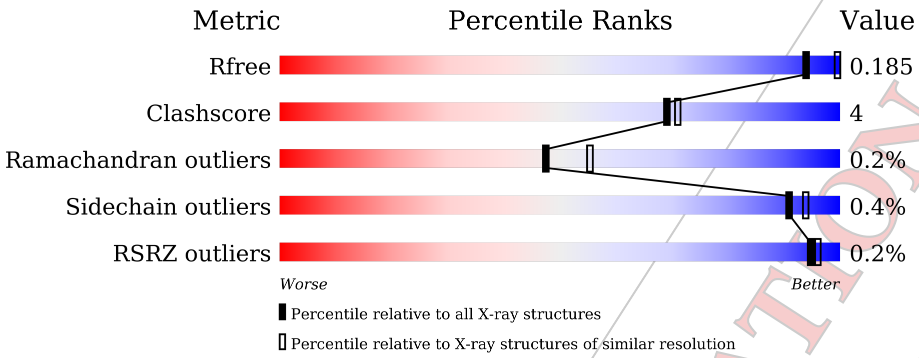

Metric	Whole archive (#Entries)	Similar resolution (#Entries, resolution range(Å))
R_{free}	130704	1377 (2.26-2.26)
Clashscore	141614	1487 (2.26-2.26)
Ramachandran outliers	138981	1449 (2.26-2.26)
Sidechain outliers	138945	1450 (2.26-2.26)
RSRZ outliers	127900	1356 (2.26-2.26)

The table below summarises the geometric issues observed across the polymeric chains and their fit to the electron density. The red, orange, yellow and green segments of the lower bar indicate the fraction of residues that contain outliers for >=3, 2, 1 and 0 types of geometric quality criteria respectively. A grey segment represents the fraction of residues that are not modelled. The numeric value for each fraction is indicated below the corresponding segment, with a dot representing fractions <=5% The upper red bar (where present) indicates the fraction of residues that have poor fit to the electron density. The numeric value is given above the bar.

Mol	Chain	Length	Quality of chain
1	A	619	84% 7% 9%

CONFIDENTIAL VALIDATION REPORT

2 Entry composition (i)

There are 4 unique types of molecules in this entry. The entry contains 9419 atoms, of which 4391 are hydrogens and 0 are deuteriums.

In the tables below, the ZeroOcc column contains the number of atoms modelled with zero occupancy, the AltConf column contains the number of residues with at least one atom in alternate conformation and the Trace column contains the number of residues modelled with at most 2 atoms.

- Molecule 1 is a protein called Lactate-binding periplasmic protein TTHA0766,Lactate-binding periplasmic protein TTHA0766.

Mol	Chain	Residues	Atoms						ZeroOcc	AltConf	Trace
			Total	C	H	N	O	S			
1	A	564	8897	2907	4386	768	821	15	0	0	0

There are 36 discrepancies between the modelled and reference sequences:

Chain	Residue	Modelled	Actual	Comment	Reference
A	-31	HIS	-	expression tag	UNP Q5SK82
A	-30	HIS	-	expression tag	UNP Q5SK82
A	-29	HIS	-	expression tag	UNP Q5SK82
A	-28	HIS	-	expression tag	UNP Q5SK82
A	-27	HIS	-	expression tag	UNP Q5SK82
A	-26	HIS	-	expression tag	UNP Q5SK82
A	-25	GLY	-	expression tag	UNP Q5SK82
A	-24	MET	-	expression tag	UNP Q5SK82
A	-23	ALA	-	expression tag	UNP Q5SK82
A	-22	SER	-	expression tag	UNP Q5SK82
A	-21	MET	-	expression tag	UNP Q5SK82
A	-20	THR	-	expression tag	UNP Q5SK82
A	-19	GLY	-	expression tag	UNP Q5SK82
A	-18	GLY	-	expression tag	UNP Q5SK82
A	-17	GLN	-	expression tag	UNP Q5SK82
A	-16	GLN	-	expression tag	UNP Q5SK82
A	-15	MET	-	expression tag	UNP Q5SK82
A	-14	GLY	-	expression tag	UNP Q5SK82
A	-13	ARG	-	expression tag	UNP Q5SK82
A	-12	ASP	-	expression tag	UNP Q5SK82
A	-11	LEU	-	expression tag	UNP Q5SK82
A	-10	TYR	-	expression tag	UNP Q5SK82
A	-9	ASP	-	expression tag	UNP Q5SK82
A	-8	ASP	-	expression tag	UNP Q5SK82
A	-7	ASP	-	expression tag	UNP Q5SK82
A	-6	ASP	-	expression tag	UNP Q5SK82

Continued on next page...

CONFIDENTIAL VALIDATION REPORT

Continued from previous page...

Chain	Residue	Modelled	Actual	Comment	Reference
A	-5	LYS	-	expression tag	UNP Q5SK82
A	-4	ASP	-	expression tag	UNP Q5SK82
A	-3	PRO	-	expression tag	UNP Q5SK82
A	-2	SER	-	expression tag	UNP Q5SK82
A	-1	SER	-	expression tag	UNP Q5SK82
A	0	ARG	-	expression tag	UNP Q5SK82
A	1	MET	-	expression tag	UNP Q5SK82
A	116	ASN	ILE	engineered mutation	UNP Q5SK82
A	138	THR	ILE	engineered mutation	UNP Q5SK82
A	142	ARG	LYS	engineered mutation	UNP Q5SK82

- Molecule 2 is CALCIUM ION (three-letter code: CA) (formula: Ca) (labeled as "Ligand of Interest" by depositor).

Mol	Chain	Residues	Atoms		ZeroOcc	AltConf
			Total	Ca		
2	A	1	1	1	0	0

- Molecule 3 is (2S)-2-HYDROXYPROPANOIC ACID (three-letter code: 2OP) (formula: $C_3H_6O_3$) (labeled as "Ligand of Interest" by depositor).

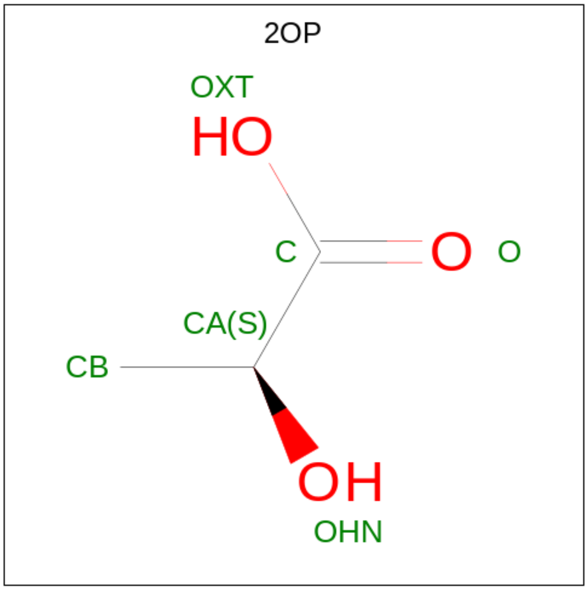

Mol	Chain	Residues	Atoms				ZeroOcc	AltConf
			Total	C	H	O		
3	A	1	11	3	5	3	0	0

- Molecule 4 is water.

CONFIDENTIAL VALIDATION REPORT

Mol	Chain	Residues	Atoms		ZeroOcc	AltConf
			Total	O		
4	A	510	510	510	0	0

CONFIDENTIAL VALIDATION REPORT

3 Residue-property plots (i)

These plots are drawn for all protein, RNA, DNA and oligosaccharide chains in the entry. The first graphic for a chain summarises the proportions of the various outlier classes displayed in the second graphic. The second graphic shows the sequence view annotated by issues in geometry and electron density. Residues are color-coded according to the number of geometric quality criteria for which they contain at least one outlier: green = 0, yellow = 1, orange = 2 and red = 3 or more. A red dot above a residue indicates a poor fit to the electron density (RSRZ > 2). Stretches of 2 or more consecutive residues without any outlier are shown as a green connector. Residues present in the sample, but not in the model, are shown in grey.

• Molecule 1: Lactate-binding periplasmic protein TTHA0766,Lactate-binding periplasmic protein TTHA0766

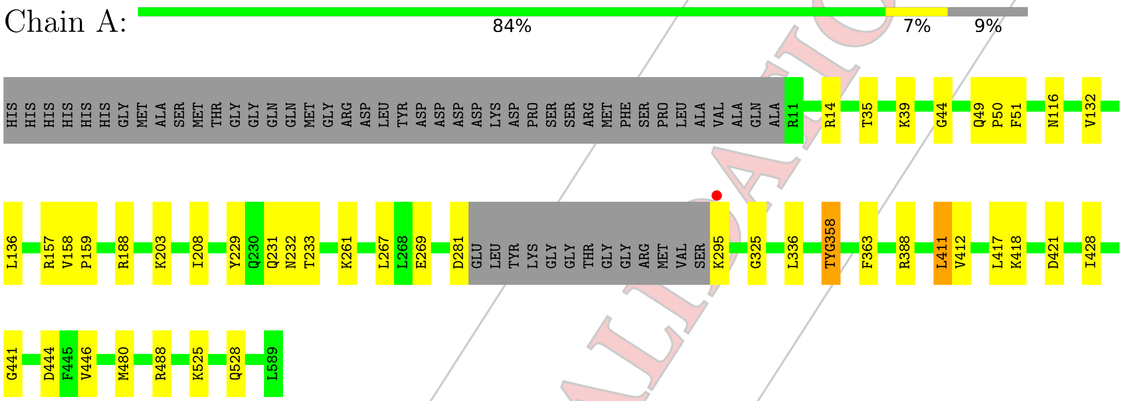

CONFIDENTIAL VALIDATION REPORT

4 Data and refinement statistics (i)

Property	Value	Source
Space group	C 2 2 21	Depositor
Cell constants<br>a, b, c, α, β, γ	102.22Å 104.98Å 138.75Å<br>90.00° 90.00° 90.00°	Depositor
Resolution (Å)	52.49 – 2.25<br>52.49 – 2.25	Depositor<br>EDS
% Data completeness<br>(in resolution range)	100.0 (52.49-2.25)<br>100.0 (52.49-2.25)	Depositor<br>EDS
R_{merge}	(Not available)	Depositor
R_{sym}	(Not available)	Depositor
$< I/\sigma(I) >$ [1]	3.57 (at 2.25Å)	Xtriage
Refinement program	PHENIX 1.14_3260	Depositor
R, R_{free}	0.147 , 0.185<br>0.147 , 0.185	Depositor<br>DCC
R_{free} test set	2005 reflections (5.61%)	wwPDB-VP
Wilson B-factor (Å^2)	19.3	Xtriage
Anisotropy	0.056	Xtriage
Bulk solvent k_{sol}(e/Å^3), B_{sol}(Å^2)	0.38 , 43.5	EDS
L-test for twinning[2]	$< \|L\| > = 0.50$, $< L^2 > = 0.34$	Xtriage
Estimated twinning fraction	0.024 for -k,-h,-l	Xtriage
F_o,F_c correlation	0.96	EDS
Total number of atoms	9419	wwPDB-VP
Average B, all atoms (Å^2)	23.0	wwPDB-VP

Xtriage's analysis on translational NCS is as follows: *The largest off-origin peak in the Patterson function is 5.19% of the height of the origin peak. No significant pseudotranslation is detected.*

[1]Intensities estimated from amplitudes.

[2]Theoretical values of $< |L| >$, $< L^2 >$ for acentric reflections are 0.5, 0.333 respectively for untwinned datasets, and 0.375, 0.2 for perfectly twinned datasets.

CONFIDENTIAL VALIDATION REPORT

5 Model quality (i)

5.1 Standard geometry (i)

Bond lengths and bond angles in the following residue types are not validated in this section: CRO, 2OP, CA

The Z score for a bond length (or angle) is the number of standard deviations the observed value is removed from the expected value. A bond length (or angle) with $|Z| > 5$ is considered an outlier worth inspection. RMSZ is the root-mean-square of all Z scores of the bond lengths (or angles).

Mol	Chain	Bond lengths		Bond angles	
		RMSZ	$\#|Z| > 5$	RMSZ	$\#|Z| > 5$
1	A	0.57	0/4610	0.72	3/6247 (0.0%)

There are no bond length outliers.

All (3) bond angle outliers are listed below:

Mol	Chain	Res	Type	Atoms	Z	Observed(^{o})	Ideal(^{o})
1	A	444	ASP	CB-CG-OD1	7.63	125.17	118.30
1	A	444	ASP	CB-CG-OD2	-7.21	111.81	118.30
1	A	411	LEU	CA-CB-CG	-5.92	101.69	115.30

There are no chirality outliers.

There are no planarity outliers.

5.2 Too-close contacts (i)

In the following table, the Non-H and H(model) columns list the number of non-hydrogen atoms and hydrogen atoms in the chain respectively. The H(added) column lists the number of hydrogen atoms added and optimized by MolProbity. The Clashes column lists the number of clashes within the asymmetric unit, whereas Symm-Clashes lists symmetry-related clashes.

Mol	Chain	Non-H	H(model)	H(added)	Clashes	Symm-Clashes
1	A	4511	4386	4400	33	2
2	A	1	0	0	0	0
3	A	6	5	4	0	0
4	A	510	0	0	13	0
All	All	5028	4391	4404	33	2

The all-atom clashscore is defined as the number of clashes found per 1000 atoms (including hydrogen atoms). The all-atom clashscore for this structure is 4.

CONFIDENTIAL VALIDATION REPORT

All (33) close contacts within the same asymmetric unit are listed below, sorted by their clash magnitude.

Atom-1	Atom-2	Interatomic distance (Å)	Clash overlap (Å)
1:A:421:ASP:OD2	4:A:701:HOH:O	1.81	0.98
1:A:203:LYS:NZ	4:A:702:HOH:O	2.10	0.84
1:A:49:GLN:HG2	1:A:51:PHE:CE2	2.18	0.79
1:A:295:LYS:N	4:A:703:HOH:O	2.16	0.79
1:A:525:LYS:NZ	4:A:704:HOH:O	2.18	0.75
1:A:188:ARG:HD2	4:A:725:HOH:O	1.90	0.71
1:A:267:LEU:HD21	1:A:269:GLU:HG3	1.72	0.70
1:A:441:GLY:HA3	4:A:905:HOH:O	1.94	0.68
1:A:261:LYS:HA	1:A:261:LYS:HE3	1.81	0.63
1:A:231:GLN:HG3	4:A:1130:HOH:O	2.01	0.60
1:A:488:ARG:NH1	4:A:717:HOH:O	2.34	0.60
1:A:417:LEU:C	1:A:417:LEU:HD23	2.28	0.53
1:A:39:LYS:HG3	1:A:44:GLY:HA2	1.92	0.51
1:A:363:PHE:CE2	1:A:411:LEU:HD22	2.47	0.50
1:A:363:PHE:HE2	1:A:411:LEU:HD22	1.78	0.49
1:A:281:ASP:OD2	4:A:705:HOH:O	2.20	0.49
1:A:261:LYS:HA	1:A:261:LYS:CE	2.42	0.48
1:A:132:VAL:HB	1:A:480:MET:HB2	1.96	0.47
1:A:136:LEU:C	1:A:136:LEU:HD12	2.35	0.47
1:A:49:GLN:HG2	1:A:51:PHE:CZ	2.51	0.46
1:A:35:THR:HG21	1:A:50:PRO:HB3	1.98	0.46
1:A:411:LEU:HG	1:A:412:VAL:N	2.29	0.45
1:A:158:VAL:HB	1:A:159:PRO:HD2	1.99	0.45
1:A:325:GLY:HA3	1:A:336:LEU:HD23	1.99	0.44
1:A:428:ILE:HD12	1:A:428:ILE:N	2.33	0.44
1:A:229:TYR:O	1:A:388:ARG:HA	2.18	0.44
1:A:231:GLN:CG	4:A:1130:HOH:O	2.64	0.43
1:A:528:GLN:OE1	4:A:706:HOH:O	2.22	0.42
1:A:233:THR:HG22	4:A:828:HOH:O	2.18	0.42
1:A:418:LYS:HE2	4:A:724:HOH:O	2.19	0.42
1:A:208:ILE:HG13	1:A:232:ASN:HB2	2.01	0.42
1:A:358:CRO:N1	1:A:358:CRO:HA31	2.35	0.42
1:A:358:CRO:HD2	1:A:358:CRO:N2	2.36	0.41

All (2) symmetry-related close contacts are listed below. The label for Atom-2 includes the symmetry operator and encoded unit-cell translations to be applied.

Atom-1	Atom-2	Interatomic distance (Å)	Clash overlap (Å)
1:A:14:ARG:HH21	1:A:229:TYR:OH[4_555]	1.37	0.23

Continued on next page...

CONFIDENTIAL VALIDATION REPORT

Continued from previous page...

Atom-1	Atom-2	Interatomic distance (Å)	Clash overlap (Å)
1:A:14:ARG:NH2	1:A:229:TYR:OH[4_555]	2.04	0.16

5.3 Torsion angles (i)

5.3.1 Protein backbone (i)

In the following table, the Percentiles column shows the percent Ramachandran outliers of the chain as a percentile score with respect to all X-ray entries followed by that with respect to entries of similar resolution.

The Analysed column shows the number of residues for which the backbone conformation was analysed, and the total number of residues.

Mol	Chain	Analysed	Favoured	Allowed	Outliers	Percentiles	
1	A	557/619 (90%)	539 (97%)	17 (3%)	1 (0%)	47	55

All (1) Ramachandran outliers are listed below:

Mol	Chain	Res	Type
1	A	446	VAL

5.3.2 Protein sidechains (i)

In the following table, the Percentiles column shows the percent sidechain outliers of the chain as a percentile score with respect to all X-ray entries followed by that with respect to entries of similar resolution.

The Analysed column shows the number of residues for which the sidechain conformation was analysed, and the total number of residues.

Mol	Chain	Analysed	Rotameric	Outliers	Percentiles	
1	A	468/514 (91%)	466 (100%)	2 (0%)	91	94

All (2) residues with a non-rotameric sidechain are listed below:

Mol	Chain	Res	Type
1	A	116	ASN
1	A	157	ARG

Sometimes sidechains can be flipped to improve hydrogen bonding and reduce clashes. There are

CONFIDENTIAL VALIDATION REPORT

no such sidechains identified.

5.3.3 RNA (i)

There are no RNA molecules in this entry.

5.4 Non-standard residues in protein, DNA, RNA chains (i)

1 non-standard protein/DNA/RNA residue is modelled in this entry.

In the following table, the Counts columns list the number of bonds (or angles) for which Mogul statistics could be retrieved, the number of bonds (or angles) that are observed in the model and the number of bonds (or angles) that are defined in the Chemical Component Dictionary. The Link column lists molecule types, if any, to which the group is linked. The Z score for a bond length (or angle) is the number of standard deviations the observed value is removed from the expected value. A bond length (or angle) with $|Z| > 2$ is considered an outlier worth inspection. RMSZ is the root-mean-square of all Z scores of the bond lengths (or angles).

Mol	Type	Chain	Res	Link	Bond lengths			Bond angles		
					Counts	RMSZ	#$\|Z\| > 2$	Counts	RMSZ	#$\|Z\| > 2$
1	CRO	A	358	1	23,23,24	2.79	4 (17%)	30,32,34	2.38	9 (30%)

In the following table, the Chirals column lists the number of chiral outliers, the number of chiral centers analysed, the number of these observed in the model and the number defined in the Chemical Component Dictionary. Similar counts are reported in the Torsion and Rings columns. '-' means no outliers of that kind were identified.

Mol	Type	Chain	Res	Link	Chirals	Torsions	Rings
1	CRO	A	358	1	-	6/12/31/32	0/2/2/2

All (4) bond length outliers are listed below:

Mol	Chain	Res	Type	Atoms	Z	Observed(Å)	Ideal(Å)
1	A	358	CRO	CA2-C2	-11.14	1.37	1.48
1	A	358	CRO	CG2-CB2	4.56	1.55	1.46
1	A	358	CRO	C2-N3	-3.57	1.31	1.39
1	A	358	CRO	CA2-N2	-2.71	1.32	1.38

All (9) bond angle outliers are listed below:

Mol	Chain	Res	Type	Atoms	Z	Observed(^{o})	Ideal(^{o})
1	A	358	CRO	CA2-C2-N3	7.99	107.15	103.37
1	A	358	CRO	O2-C2-CA2	-6.46	127.33	130.96

Continued on next page...

CONFIDENTIAL VALIDATION REPORT

Continued from previous page...

Mol	Chain	Res	Type	Atoms	Z	Observed(°)	Ideal(°)
1	A	358	CRO	C1-CA1-N1	-3.40	104.45	109.96
1	A	358	CRO	O3-C3-CA3	-3.26	116.53	126.39
1	A	358	CRO	C2-N3-C1	-3.07	106.41	107.97
1	A	358	CRO	CA1-C1-N3	-2.97	121.18	124.75
1	A	358	CRO	CB1-CA1-N1	-2.66	96.21	113.84
1	A	358	CRO	CG2-CB2-CA2	-2.48	126.91	129.94
1	A	358	CRO	CA1-C1-N2	2.12	126.85	123.89

There are no chirality outliers.

All (6) torsion outliers are listed below:

Mol	Chain	Res	Type	Atoms
1	A	358	CRO	N1-CA1-CB1-CG1
1	A	358	CRO	N1-CA1-CB1-OG1
1	A	358	CRO	C1-CA1-CB1-CG1
1	A	358	CRO	C1-CA1-CB1-OG1
1	A	358	CRO	N3-C1-CA1-CB1
1	A	358	CRO	N2-C1-CA1-CB1

There are no ring outliers.

1 monomer is involved in 2 short contacts:

Mol	Chain	Res	Type	Clashes	Symm-Clashes
1	A	358	CRO	2	0

5.5 Carbohydrates (i)

There are no monosaccharides in this entry.

5.6 Ligand geometry (i)

Of 2 ligands modelled in this entry, 1 is monoatomic - leaving 1 for Mogul analysis.

In the following table, the Counts columns list the number of bonds (or angles) for which Mogul statistics could be retrieved, the number of bonds (or angles) that are observed in the model and the number of bonds (or angles) that are defined in the Chemical Component Dictionary. The Link column lists molecule types, if any, to which the group is linked. The Z score for a bond length (or angle) is the number of standard deviations the observed value is removed from the expected value. A bond length (or angle) with $|Z| > 2$ is considered an outlier worth inspection. RMSZ is the root-mean-square of all Z scores of the bond lengths (or angles).

CONFIDENTIAL VALIDATION REPORT

Mol	Type	Chain	Res	Link	Bond lengths			Bond angles		
					Counts	RMSZ	#|Z| > 2	Counts	RMSZ	#|Z| > 2
3	2OP	A	602	2	2,5,5	0.32	0	3,6,6	0.81	0

In the following table, the Chirals column lists the number of chiral outliers, the number of chiral centers analysed, the number of these observed in the model and the number defined in the Chemical Component Dictionary. Similar counts are reported in the Torsion and Rings columns. '-' means no outliers of that kind were identified.

Mol	Type	Chain	Res	Link	Chirals	Torsions	Rings
3	2OP	A	602	2	-	0/0/4/4	-

There are no bond length outliers.

There are no bond angle outliers.

There are no chirality outliers.

There are no torsion outliers.

There are no ring outliers.

No monomer is involved in short contacts.

5.7 Other polymers (i)

There are no such residues in this entry.

5.8 Polymer linkage issues (i)

There are no chain breaks in this entry.

CONFIDENTIAL VALIDATION REPORT

6 Fit of model and data (i)

6.1 Protein, DNA and RNA chains (i)

In the following table, the column labelled '#RSRZ> 2' contains the number (and percentage) of RSRZ outliers, followed by percent RSRZ outliers for the chain as percentile scores relative to all X-ray entries and entries of similar resolution. The OWAB column contains the minimum, median, 95th percentile and maximum values of the occupancy-weighted average B-factor per residue. The column labelled 'Q< 0.9' lists the number of (and percentage) of residues with an average occupancy less than 0.9.

Mol	Chain	Analysed	<RSRZ>	#RSRZ>2			OWAB(Å^2)	Q<0.9
1	A	563/619 (90%)	-0.44	1 (0%)	95	96	9, 19, 37, 61	0

All (1) RSRZ outliers are listed below:

Mol	Chain	Res	Type	RSRZ
1	A	295	LYS	2.8

6.2 Non-standard residues in protein, DNA, RNA chains (i)

In the following table, the Atoms column lists the number of modelled atoms in the group and the number defined in the chemical component dictionary. The B-factors column lists the minimum, median, 95th percentile and maximum values of B factors of atoms in the group. The column labelled 'Q< 0.9' lists the number of atoms with occupancy less than 0.9.

Mol	Type	Chain	Res	Atoms	RSCC	RSR	B-factors(Å^2)	Q<0.9
1	CRO	A	358	22/23	0.97	0.09	11,19,29,31	0

6.3 Carbohydrates (i)

There are no monosaccharides in this entry.

6.4 Ligands (i)

In the following table, the Atoms column lists the number of modelled atoms in the group and the number defined in the chemical component dictionary. The B-factors column lists the minimum, median, 95th percentile and maximum values of B factors of atoms in the group. The column labelled 'Q< 0.9' lists the number of atoms with occupancy less than 0.9.

Mol	Type	Chain	Res	Atoms	RSCC	RSR	B-factors(Å^2)	Q<0.9
3	2OP	A	602	6/6	0.97	0.15	7,12,17,17	0

Continued on next page...

CONFIDENTIAL VALIDATION REPORT

Continued from previous page...

Mol	Type	Chain	Res	Atoms	RSCC	RSR	B-factors($Å^2$)	Q<0.9
2	CA	A	601	1/1	1.00	0.09	9,9,9,9	0

6.5 Other polymers ⓘ

There are no such residues in this entry.

CONFIDENTIAL VALIDATION REPORT
