# Supplementary material for: A genetically encoded fluorescent biosensor for extracellular l-lactate
Source: Nat Commun. 2021 Dec 6;12:7058. doi: 10.1038/s41467-021-27332-2 (PMC8648760; doi:10.1038/s41467-021-27332-2)
Supplement: Supplementary file 2 — Reporting Summary [file 41467_2021_27332_MOESM2_ESM.pdf]

nature portfolio

Corresponding author(s): Robert E. Campbell

Last updated by author(s): Nov 11, 2021

Reporting Summary

Nature Portfolio wishes to improve the reproducibility of the work that we publish. This form provides structure for consistency and transparency in reporting. For further information on Nature Portfolio policies, see our Editorial Policies and the Editorial Policy Checklist.

Statistics

For all statistical analyses, confirm that the following items are present in the figure legend, table legend, main text, or Methods section.

n/a	Confirmed	
☐	☒	The exact sample size (*n*) for each experimental group/condition, given as a discrete number and unit of measurement
☐	☒	A statement on whether measurements were taken from distinct samples or whether the same sample was measured repeatedly
☐	☒	The statistical test(s) used AND whether they are one- or two-sided<br>*Only common tests should be described solely by name; describe more complex techniques in the Methods section.*
☒	☐	A description of all covariates tested
☐	☒	A description of any assumptions or corrections, such as tests of normality and adjustment for multiple comparisons
☐	☒	A full description of the statistical parameters including central tendency (e.g. means) or other basic estimates (e.g. regression coefficient) AND variation (e.g. standard deviation) or associated estimates of uncertainty (e.g. confidence intervals)
☐	☒	For null hypothesis testing, the test statistic (e.g. *F*, *t*, *r*) with confidence intervals, effect sizes, degrees of freedom and *P* value noted<br>*Give P values as exact values whenever suitable.*
☒	☐	For Bayesian analysis, information on the choice of priors and Markov chain Monte Carlo settings
☒	☐	For hierarchical and complex designs, identification of the appropriate level for tests and full reporting of outcomes
☒	☐	Estimates of effect sizes (e.g. Cohen's *d*, Pearson's *r*), indicating how they were calculated

Our web collection on statistics for biologists contains articles on many of the points above.

Software and code

Policy information about availability of computer code

Data collection	We used cellSens Dimension (version 2.3), MatLab (2013) running open source scanning microscope control software ScanImage (version 3.81, HHMI/Janelia Research Campus), Phenix (version 1.19-4092), COOT (version 0.8.9.2) and NIS-Elements AR (4.60.00 64-bit) softwares to run our microscopes and data acquisition modules.
Data analysis	Data analysis was performed by Excel (versions 16.50), ImageJ (Fiji version 1.0), Igor Pro (version 8.04) and Prism (GraphPad Software). Custom code is available from https://github.com/shucez/eLACCO_manuscript_TIRF_deltaF_F0.

For manuscripts utilizing custom algorithms or software that are central to the research but not yet described in published literature, software must be made available to editors and reviewers. We strongly encourage code deposition in a community repository (e.g. GitHub). See the Nature Portfolio guidelines for submitting code & software for further information.

Data

Policy information about availability of data

All manuscripts must include a data availability statement. This statement should provide the following information, where applicable:

- Accession codes, unique identifiers, or web links for publicly available datasets
- A description of any restrictions on data availability
- For clinical datasets or third party data, please ensure that the statement adheres to our policy

Structure coordinates of eLACCO1 have been deposited in the Protein Data Bank with a code of 7E9Y (https://www.rcsb.org/structure/unreleased/7E9Y). The plasmids have been deposited to Addgene (https://www.addgene.org/browse/article/28216216/ and https://www.addgene.org/browse/article/28216308/). Source data are provided with this paper.

Field-specific reporting

Please select the one below that is the best fit for your research. If you are not sure, read the appropriate sections before making your selection.

- [x] Life sciences
- [ ] Behavioural & social sciences
- [ ] Ecological, evolutionary & environmental sciences

For a reference copy of the document with all sections, see nature.com/documents/nr-reporting-summary-flat.pdf

Life sciences study design

All studies must disclose on these points even when the disclosure is negative.

Sample size	Sample size was estimated based on the previous published studies in the development of biosensors. [1] Shen Y., et al. "Genetically encoded fluorescent indicators for imaging intracellular potassium ion concentration." Communications Biology 2, 18 (2019). [2] Qian Y., et al. "A genetically encoded near-infrared fluorescent calcium ion indicator" Nature Methods 16, 171-174 (2019).
Data exclusions	No data were excluded.
Replication	All experiments were replicated. The replication number of each experiment is described in the caption of corresponding figures. Results were repeatable in all conditions.
Randomization	Samples were randomly allocated into groups.
Blinding	Experimenters were not blinded to the group allocation. Blinding was not required since the experimental conditions were obvious to the researchers and all transfected cells were identified and analyzed by unbiased statistical tests.

Reporting for specific materials, systems and methods

We require information from authors about some types of materials, experimental systems and methods used in many studies. Here, indicate whether each material, system or method listed is relevant to your study. If you are not sure if a list item applies to your research, read the appropriate section before selecting a response.

Materials & experimental systems

n/a	Involved in the study	
x		Antibodies
	x	Eukaryotic cell lines
x		Palaeontology and archaeology
	x	Animals and other organisms
x		Human research participants
x		Clinical data
x		Dual use research of concern

Methods

n/a	Involved in the study	
x		ChIP-seq
x		Flow cytometry
x		MRI-based neuroimaging

Eukaryotic cell lines

Policy information about cell lines

Cell line source(s)	HeLa cell line (originally purchased from ATCC) was a kind gift from Prof. Takeaki Ozawa, The University of Tokyo. HEK293FT cell line was sourced from Thermo Fisher Scientific (catalog #R70007). T98G cell line was sourced from ATCC.
Authentication	No authentication was performed as the cell lines come from reliable sources.
Mycoplasma contamination	All cell lines were negative for mycoplasma contamination.
Commonly misidentified lines (See ICLAC register)	No commonly misidentified cell lines were used in this study.

Animals and other organisms

Policy information about studies involving animals; ARRIVE guidelines recommended for reporting animal research

Laboratory animals	Male mice (C57bl/6, P45 days old) were brought to the lab on the day of the experiment. Housing condition for mice is room temperature, standard 12hr/12hr light/dark cycle, ad libitum access to food and water, and 50-70% humidity.
Wild animals	No wild animals were used in this study.

Field-collected samples	The study did not involved samples collected in the field.
Ethics oversight	For experiments performed at University of Calgary, all methods for animal care and use were approved by the University of Calgary Animal Care and Use Committee and were in accordance with the National Institutes of Health Guide for the Care and Use of Laboratory Animals. For experiments at HHMI Janelia Research Campus, all surgical and experimental procedures were in accordance with protocols approved by the HHMI Janelia Research Campus Institutional Animal Care and Use Committee and Institutional Biosafety Committee.

Note that full information on the approval of the study protocol must also be provided in the manuscript.
